# The immunological benefit of higher dose N-acetyl cysteine following mechanical ventilation in critically ill patients

**DOI:** 10.1186/2008-2231-22-57

**Published:** 2014-07-15

**Authors:** Atabak Najafi, Mojtaba Mojtahedzadeh, Keyvan Haji Ahmadi, Mohammad Abdollahi, Maryam Mousavi, Legese Chelkeba, Farhad Najmeddin, Arezoo Ahmadi

**Affiliations:** 1 Department of Anesthesiology and Critical Care Medicine, Sina Hospital, Faculty of Medicine, Tehran University of Medical Sciences, Tehran, Iran; 2 Department of Clinical Pharmacy, Faculty of Pharmacy, Tehran University of Medical Sciences, Tehran, Iran; 3 Faculty of Pharmacy and Pharmaceutical Sciences Research Center, Tehran University of Medical Sciences, Tehran, Iran; 4 Department of Clinical Pharmacy, Faculty of Pharmacy, Mashhad University of Medical sciences, Mashhad, Iran

**Keywords:** N-Acetyl, Cysteine (NAC), Glutathione (GSH), Human β Defensin 2 (HβD2), Immunoglobulin M (IgM), Multiple trauma, Sepsis

## Abstract

**Background:**

Sepsis complication is a major cause of death in multiple trauma critically ill patients. Defensin (cysteine rich anti-microbial peptides), as an important component of immune system, might play an important role in this process. There is also rising data on immunological effects of N-acetyl-cysteine (NAC), a commonly used anti-oxidant in oxidative stress conditions and glutathione (GSH) deficiencies. The aim of the present study was to evaluate the potential beneficial effects of NAC administration on multiple trauma patients with sepsis.

**Methods:**

In a prospective, randomized controlled study, 44 multiple trauma critically ill patients who were mechanically ventilated and met the criteria of sepsis and admitted to the intensive care unit (ICU) were randomized into two groups . Control group received all standard ICU therapies and NAC group received intravenous NAC 3 gr every 6 hours for 72 hours in addition to standard therapies. Acute Physiology and Chronic Health Evaluation II (APACHE II) and Sequential Organ Failure Assessment (SOFA) scores, length of ICU stay, ICU mortality were recorded. Levels of serum Immunoglobulin M (IgM), Human β-Defensin 2 (HβD2) and GSH were assessed at baseline and 24, 72, 120 hours after intervention.

**Results:**

During a period of 13-month screening, 44 patients underwent randomization but 5 patients had to be excluded. 21 patients in NAC group and 18 patients in control group completed the study. For both groups the length of ICU stay, SOFA score and systemic oxygenation were similar. Mortality rate (40% vs. 22% respectively, p = 0.209) and ventilator days (Mean ± SD 19.82 ± 19.55 days vs. 13.82 ± 11.89 days respectively, p = 0.266) were slightly higher for NAC group. IgM and GSH levels were similar between two groups (p = 0.325, 0.125 respectively), HβD2 levels were higher for NAC group (at day 3).

**Conclusion:**

High dose of NAC administration not only did not improve patients’ outcome, but also raised the risk of inflammation and was associated with increased serum creatinine.

## Background

Multiple trauma, a complex syndrome of injuries to various anatomical regions with defined intensity and subsequent systemic reactions [1]. Trauma is the major cause of death before the age of 45 in developed countries [2,3], claiming over 6 million kills annually around the world which is preventable in approximately 20% of cases [4,5]. Three pre-eminent causes of death in this patients are head trauma, hemorrhage and sepsis/Multi Organ Failure (MOF) [6]. Tissue ischemia induces Reactive Oxygen Species (ROS) production which in turn activates neutrophils and attracts them to the injury site. Extensive progression of this and other similar processes following trauma insult enhance the local inflammatory response to remote organs, resulting in the development of Systemic Inflammatory Response Syndrome (SIRS) [7-12]. In sepsis, acute immunologic response and excessive release of pro-inflammatory cytokines [Interleukin 1(IL-1), Tumor Necrosis Factor α (TNF-α)] trigger target cells (leukocytes, endothelial cells, hepatocytes, lung and intestine epithelium) and induce cytokine, chemokine, ROS and proteolytic enzymes production [13-16].

It is obvious that patients with sepsis or multiple trauma require mechanical ventilation support. Due to the high concentration of inspired oxygen used, the probability of alveolar epithelial damage is high as the result of formation of free oxygen radicals. Some studies have suggested that the use of high doses of antioxidants in these patients could be beneficial [7].

Defensins are cysteine rich endogenous antimicrobial peptides which consist of to approximately 5% of total granulocyte proteins, following infection their levels in systemic circulation increases, defensins have a significant effect on a wide spectrum of microorganisms [17-19] and beyond that they also act as immunologic mediators [20]. Human β Defensin 2 (HβD2) is an inducible defensin, whose induction is impaired during sepsis [20,21].

NAC is a well-known anti-oxidant which is administered in various conditions where oxidative stress is present [22-24]. NAC is thiol containing compound that in addition to direct radical scavenging properties is a precursor for glutathione (GSH) synthesis [23,24]. NAC seems to have immunological effects (doses higher than 12 gram/day) that can affect different cytokines (IL-2, IL-4, IL-6, IL-10, TNF-α, NF-κB) [25,26] and phagocytic function [27]. NAC has received so much credit for its nephro-protective properties when used prior to radio-contrast media administration. The ischemia compounded by increased medullary O_2_consumption may somewhat resemble ischemic acute tubular necrosis (ATN) in trauma and sepsis. Although near 10 Meta-Analyses has been published to justify its use many of these studies have significant pitfalls [25-27].

To our knowledge there are no studies about the effect of NAC as an antioxidant and/or immunomodulator in patients with SIRS/sepsis criteria following multiple trauma. Therefore, this study aimed to investigate HβD2 levels in multiple trauma patients with SIRS/sepsis and its potential correlation with progress and outcome of therapy, and to evaluate the effects of high dose NAC administration, in the early onset of sepsis during first hours to days after trauma, on immunologic responses and overall outcome. Furthermore, this study sought to examine the hypothesis that NAC administration can alter HβD2 levels either by immuno-modulatory activity or as a cysteine source.

## Methods

### Study design and patient population

Between March 2011 to April 2012, this prospective randomized clinical trial was conducted in general ICU of "Sina"and "Imam Khomeini" Hospital of Tehran University of Medical Sciences (TUMS), Iran. In all cases, informed consents were received from their legal guardian. The study protocol was approved by the ethical committee of TUMS. Our clinical trial was registered in Iranian Registry of Clinical Trial with code number of (IRCT 2014010716120 N1).

All the patients enrolled in the study had multiple trauma. The inclusion criteria were age older than 18 years, mechanical ventilation with FiO_2_ more than 50%, an Acute Physiology and Chronic Health Evaluation (APACHE) II score greater than 15, suspected infection and at least two Systemic Inflammatory Response (SIRS) criteria (defined as the following conditions: body temperature > 38°C or < 36°C, heart rate > 90 beats/min, respiratory rate > 20 breath/min or Paco_2_ < 32 mmHg, WBC >12,000 cells/mm^3^, or < 4000 cells/mm^3^, or > 10% immature [band] cells) diagnosed during the first 24 hours of ICU admission. Exclusion criteria for all patients were ages below 18 years, pregnancy, breast feeding, beyond 24 hours of sepsis diagnosis, Central Venous Pressure (CVP) > 18 mmHg, Systolic Blood Pressure < 90 mmHg or Mean Arterial Pressure (MAP) < 60 mmHg, Serum Creatinine ≥ 2 mg/dl, liver dysfunction (defined as the presence of hepatic cirrhosis or concentration of serum transaminases greater than third times the upper limit of normal range), history of myocardial infarction or heart failure (EF < 40%) and those received N-acetyl Cysteine (NAC) before initiation of study.

**Figure 1:**
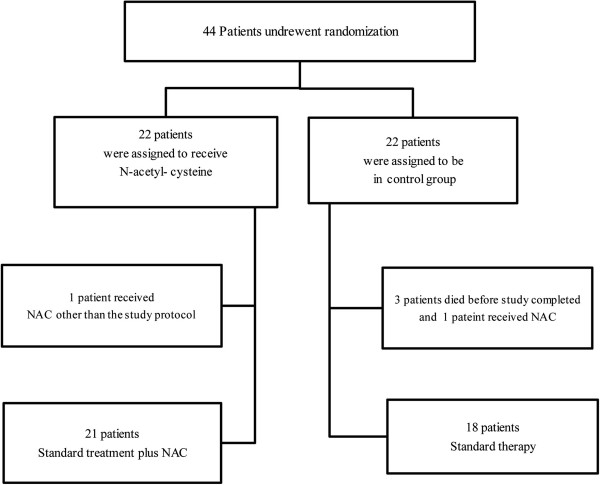
Enrollment and protocol study.

Patients were randomized via block randomization to receive one of the following treatments: standard treatment for sepsis (control group) and standard treatment plus NAC (intervention group) for 72 hours. Standard treatments for sepsis were implemented as following: early resuscitation within the first 6 hours of admission, appropriate diagnostic studies to ascertain causative organisms before starting antibiotics, early administration of broad-spectrum antibiotic therapy, reassessment of antibiotic therapy with microbiology and clinical data to narrow coverage, appropriate Volume resuscitation or vasopressor therapy, Mechanical ventilation adjusted to maintain SaO_2_ > 95%, PaO_2_ > 90 mmHg and PaCO_2_ between 38 and 42 mmHg, appropriate analgesia and sedation provided for all patients, insulin treatment was administered to maintain glucose at < 200 mg/dl and appropriate prophylactic measures for deep vein thrombosis (DVT) and stress-related mucosal damage (SRMD). Patients in intervention group received intravenous NAC (Exir Pharmaceutical company, Tehran, Iran) 3gr in 250 ml 5% dextrose over 30 min every 6 hours for the first 72 hours of admission (Figure 1).

Demographic data and clinical information were obtained at the beginning. Monitoring parameters including MAP, heart rate, respiratory rate and CVP of each patient were recorded in the ICU flow sheets every 3 hours. Laboratory data including serum creatinine, Blood Urea Nitrogen (BUN), hemoglobin, platelet and white blood cell (WBC) counts, albumin, sodium and potassium were also collected daily during 5 days of study period.

### Measurements

All patients had central venous catheter and arterial catheter for blood sampling. For all patients there were four scheduled time points to assess the level of IgM, GSH and HβD2. Blood samples were collected at baseline and 24, 72 and 120 hours after intervention and centrifuged at 3000 × g for 15 minutes to remove cells and cellular debris. The plasma stored at -80°C until the time of analysis. Levels of IgM, GSH and HβD2 were measured by commercially available enzyme-linked immunosorbent assay (ELISA) kits (CUSABIO® biotech co LTD, Wuhan Hubei, China) according to manufacturer instruction.

APACHE score was measured at the beginning and Sequential organ failure assessment (SOFA) score was calculated daily for 5 days. Length of ICU stay, ICU mortality and ventilator free days were recorded for all patients.

### Statistical Analysis

All data were expressed as Mean ± SD. The unpaired *t* test for parametric data and Mann–Whitney test for nonparametric data were used to compare the differences between the treatment groups at each time point. Comparisons of the differences between the time points in each treatment group, repeated-measure one-way analysis of variance and nonparametric analysis of variance (Friedman test) were performed to analyze changes in levels of the biomarkers. The paired *t* test for parametric data and Wilcoxon rank sum test for nonparametric data were done to analyze changes in variables with 2 time points (at baseline and 24 hours after). P values less than 0.05 were considered statistically significant.

## Results

**Figure 2:**
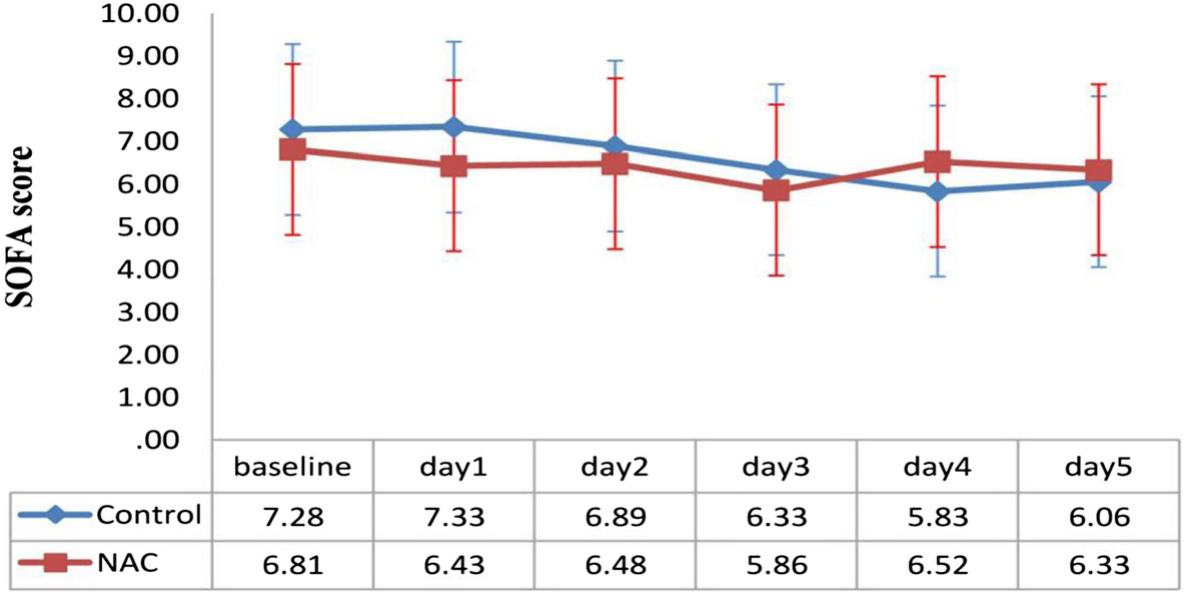
**Changes of patients SOFA score in different days of study.** Difference between two groups is not significant (P=0.708). Figure shows mean SOFA score±SE.

Between March 2011 to April 2012, 44 consecutive patients who met the inclusion criteria were enrolled in the study. Five patients excluded from the study, three of them died before the end of the study and the other 2 patients received NAC dosage other than the study protocol. The remaining 39 patients were randomly assigned to NAC group and control group, each with 21 and 18 patients respectively. Demographic and baseline values for APACHE and SOFA scores have been summarized in Table 1. The differences in age and sex were not significant between two groups. (p = 0.450 and 0.204 respectively). Differences in baseline values for APACHE and SOFA scores were not statistically significant either (Table 1). Also the patients in each group were similar at baseline regarding to heart rate, MAP, temperature and Pao_2_/Fio_2_ (Table 1).In comparison between two groups, in NAC group, ventilator days were longer (19.82 ± 19.55 vs. 13.82 ± 11.69 days), length of ICU stay was shorter (21.2 ± 20.71 vs. 27.72 ± 39.46 days) and mortality rate was higher (40% versus 22%), although the differences in ventilator days, length of ICU stay and mortality rate were not statistically significant (p = 0.266, p = 0.545, p = 0.209 respectively). Moreover, SOFA scores are associated with a trend towards a reduction in NAC group specially on day 3 but the trend was not statistically significant (Figure 2).

**Table 1:** Demographic and baseline clinical characteristic of patients

	**Control group**	**NAC group**	**P value**
**Age (years)**	44.44 ± 17.684	40.14 ± 17.828	0.450
**Male number**	15	20	0.204
**APACHE II**	20.06 ± 5.162	21.05 ± 512	0.476
**SOFA**	7.28 ± 2.109	6.86 ± 1.457	0.468
**GCS**	7.28 ± 1.934	6.55 ± 1.143	0.145
**MAP(mmHg)**	92.72 ± 17.376	93.39 ± 14.467	0.899
**HR(beat/min)**	89.94 ± 18.005	97.09 ± 19.079	0.234
**Temperature(°C)**	37.339 ± 1.01	37.145 ± 0.965	0.536
**PaO2/FiO2**	196.607 ± 105.504	219.594 ± 123.307	0.529
**SCr(mg/dl)**	1.046±	1.053 ± 0.373	0.945

NAC: N-Acetylcysteine, APACHE: Acute Physiology and Chronic Health Evaluation, SOFA: Sequential Organ Failure Assessment, GCS: Glasgow Coma Scale, MAP: Mean Arterial Pressure, HR: Heart Rate.

Data shown as Mean ± SD. P values <0.05 consired significant.

**Figure 3:**
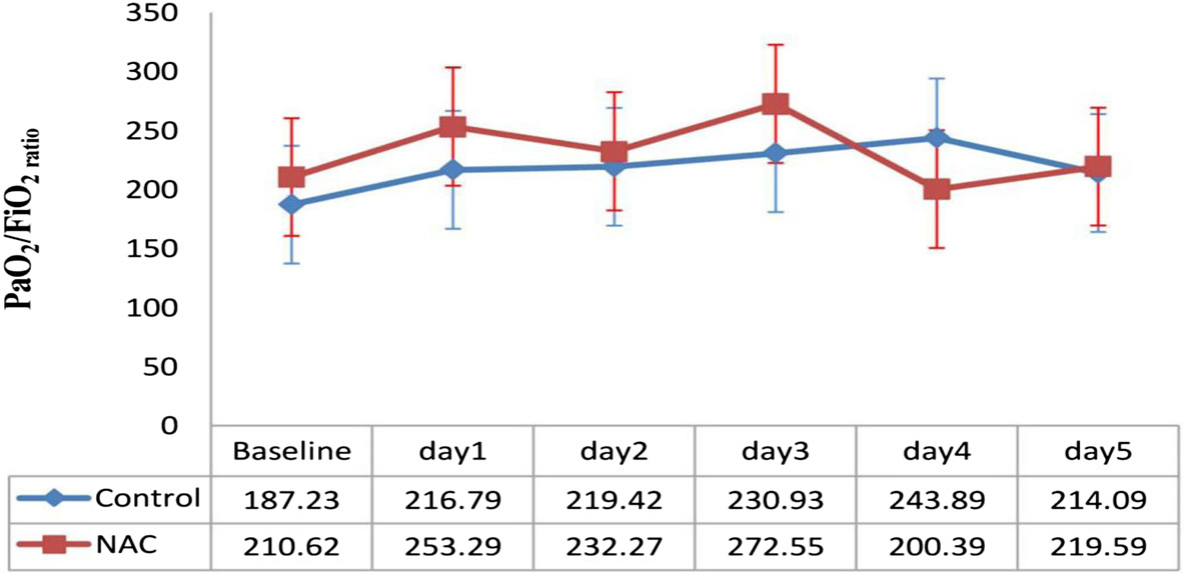
**Changes of Pao**_**2**_**/Fio**_**2 **_**ratio in patients in different days of study.** Difference between two groups is not significant (P = 0.567). Figure shows mean ±SE.

Pao_2_:Fio_2_ ratio improved in both groups (specially in the NAC group on day 3), although the difference was not significant (p = 0.567) (Figure 3). An upward trend in MAP, heart rate, WBC counts and Absolute Neutrophil Count (ANC) were shown in NAC group on day 1 through 5 after the start of NAC (Table 2). By means of paired *t*-test (before-after analysis) method, we had a significant decline in mean serum creatinine level between 1–3 days in control group (1.04 ± 0.25 versus 0.891 ± 0.253 p = 0.019). The mean serum creatinine level rose on day 1 through 3 in NAC group, however; this increase was not statistically significant (1.05 ± 0.373 versus 1.48 ± 2.87, p = 0.514). On day 5, creatinine levels reached similar levels in both groups (0.851 ± 0.205 Control group versus 0.920 ± 0.385 NAC group p = 0.502).

**Table 2:** Changes of patients HR, MAP, WBC count, ANC in different days of study

		**Base line**	**Day 1**	**Day 2**	**Day 3**	**Day 4**	**Day 5**	**P. value**
**HR**	Control	89.94	90.67	87.89	84.67	86.56	89.00	0.040
	Intervention	96.43	97.19	97.81	100.86	97.67	94.19	
**MAP**	Control	91.18	80.29	87.47	85.12	83.71	82.53	0.042
	Intervention	94.48	89.29	92.05	94.33	90.95	89.95	
**WBC count**	Control	11726	10597	8930	7890	8117	89	0.06
	Intervention	13094	11791	10165	10874	11087	11191	
**ANC**	Control	10306.07	7737.73	7425.16	5804.00	5569.42	6732.95	0.001
	Intervention	10261.22	9507.84	8468.47	9800.79	9754.49		

MAP: Mean Arterial Pressure, HR: Heart Rate, ANC: Absolute Neutrophil Count.

**Figure 4:**
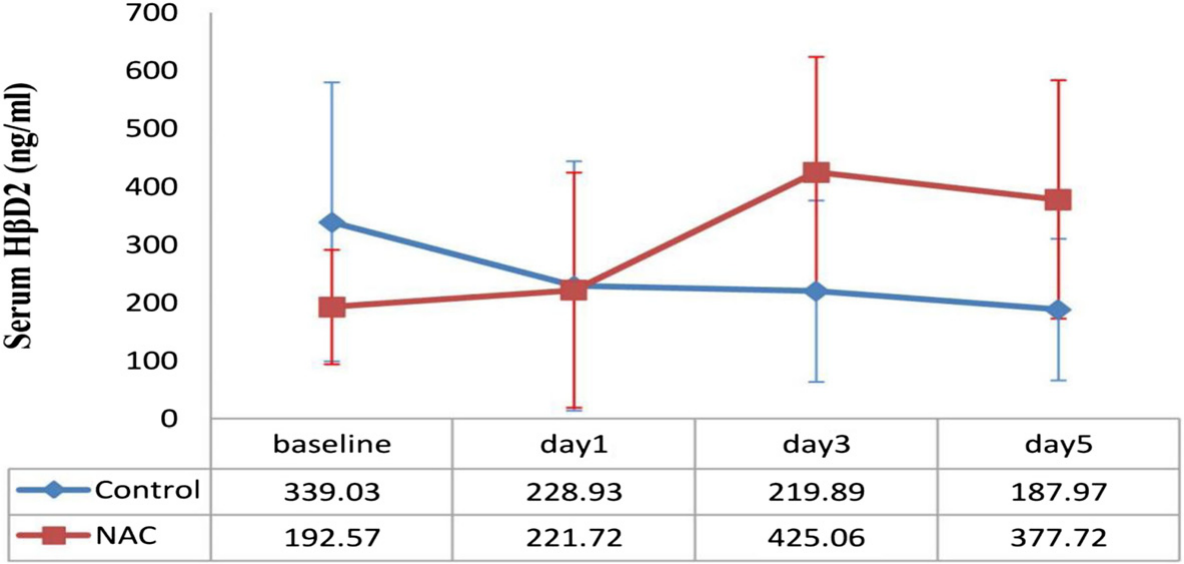
**Changes in Serum HβD**_**2 **_**in different days of study.** Difference between two groups is not significant (P = 0.463). Figure shows mean±SE.

Furthermore the changes in the level of IgM (p = 0.325) and GSH (p = 0.125) were not significant among two groups but the mean serum levels of GSH were higher in NAC group (Table 3). Despite a non significant difference in HβD2 levels between two groups (p = 0.463), HβD2 levels are associated with a trend towards an increase in NAC group specially on day 3 (Figure 4).

**Table 3:** Changes of patients IgM and GSH level in different days of study

		**Base line**	**Day1**	**Day3**	**Day5**
**IgM(mg/dl)**	Control	35.28 ± 8.67	34.59 ± 8.87	32.00 ± 2.69	34.43 ± 5.69
	NAC	34.22 ± 6.38	33.35 ± 6.80	32.18 ± 6.28	31.64 ± 3.93
**GSH(μg/dl)**	Control	11.89 ± 8.95	11.05 ± 9.52	10.99 ± 7.20	9.26 ± 6.76
	NAC	15.9 ± 9.55	14.79 ± 12.89	15.35 ± 9.51	13.51 ± 6.76

NAC: N-AcetylCysteine, IgM: Immunoglobulin M, GSH: Gluthathione, Data are shown as Mean ± SD.

## Discussion

The aim of the current study was to focus on potential immunological effects of NAC administration in critical illness, we have also reassessed the antioxidant properties which had already been well established. In previous study, immunological effects of NAC were seen with doses relatively higher than usual dosage for anti-oxidant effects (higher than 9 gram/day) [25-27]. So we have administered 12 gram/day of NAC in our study. The previous study [24] reviewed the effects of NAC in a condition of GSH deficiency such as pulmonary disease and oxidative stress, they concluded that oral administration of NAC was a safe and beneficial supplement in setting of GSH deficiency. In other study conducted on piglets, it was observed that NAC has no significant effect on the total GSH level [28]. Another study investigated the effects of NAC (150 mg/kg bolus and then 12 mg/kg/day as continuous infusion for 3–5 days) on total antioxidant potential in 60 critically ill patients [29]. The need for inotropic support and mechanical ventilation and ICU stay did not vary significantly between NAC and placebo group. Also levels of total antioxidant potential remain unchanged during intervention. Other study evaluates the effects of NAC (150 mg/kg bolus followed by a continuous infusion of 12 mg/kg/hr from a minimum of 3 days up to a maximum of 5 days) for prevention of multisystem organ failure [30]. The mortality, the required days of inotropic support, mechanical ventilation, and intensive care did not significantly change between two groups. Furthermore, patients admitted after 24 hrs of hospital admission had a significantly worse mortality in the NAC group as well. Indeed, during 10 years ago several clinical randomized trials suggested that the beneficial impact of NAC were achieved if given early, due to attenuating inflammatory response by free radical scavenging effects, but if commenced in a later phase of sepsis, it may also worsen outcome, as a result of the impaired granulocyte – dependent bacterial killing [30-32]. Nevertheless, prophylactic NAC treatment failed to prevent postoperative organ dysfunction following major abdominal surgery [33].

The results of this study show that high doses of NAC (12 gram/day) for the first 72 hours in mechanically ventilated multiple trauma patients with sepsis patients (patients with sepsis diagnosis over 24 hour was excluded), do not significantly change in the total serum GSH level. Meanwhile in NAC group, the mean level of GSH is higher than control group, it is not along with any improvement in outcome. The ventilator day and mortality rate are higher in NAC group and it shows a negative impact on HR and MAP as well. Our data suggests reconsideration and conducts large sized trials in order to evaluate the efficacy of NAC as an anti-oxidant and a thiol group supplement for GSH deficiency. Further studies are recommended to conduct with focus on commencing NAC treatment in an earlier phase of sepsis, dosage and safety issues. In our study we have observed that NAC, which is often considered as a safe and well tolerated entity, is not as safe. Furthermore, in some circumstances its indication is questionable and present data are not satisfactory.

We have assessed systemic oxygenation and respiratory functions in terms of changes in PaO_2_/FiO_2_ ratio and the number of ventilator days. In other study administered 190 mg/kg/day NAC as continuous infusion for 3 days in 42 patients with established Acute Respiratory Distress Syndrome (ARDS) and compared it with placebo [34]. The mortality rate, the need for ventilator support and ICU stay and PaO2/FiO2 ratio did not differ significantly between both groups. In another randomized double-blind, placebo controlled clinical trial by Bernard et al. NAC 70 mg/kg every 8 hours was administered for ARDS patients for 10 days [35]. The number of days of acute lung injury decreased in NAC group, yet mortality rate did not change significantly between groups. In our study we encounter similar results. The analyses show no significant difference on day 1 through 5. Improvement in Pao2/Fio2 ratio is seen on day 5 in both groups. The number of ventilator days is longer for NAC group although the difference is not statistically significant. We can suggest that routine administration high dose of NAC has no effect on ventilator days and can be harmful.

Various studies have investigated the potential effect of NAC on immune system function in different setting (animal and human studies) and focused on diverse pathological conditions (ARDS, SIRS or sepsis, hepatic injury and etc.). For instance, in other study in critically ill patients with SIRS or sepsis noticed that, high doses of NAC (12, 18 gram/day) resulted in significantly improved phagocytic activity of neutrophils as compared with low doses of NAC. In contrast, they found that high dose NAC decreases respiratory burst and leads to impaired clearance of microorganisms [27].

Santiago and co-workers investigated NAC’s effects on inflammatory response and reported that plasma IL-4 and IL-10 (anti-inflammatory cytokines) levels were higher in NAC treated group with respect to time of hepatic reperfusion, indicating a positive modulation effect of NAC on the anti inflammatory response mediated by this cytokines [25]. In in-vitro settings, NAC with similar concentrations above 25 mmol/L completely inhibited NF-κB signal. The trend was the same in in-vivo part of the study, Paterson and colleagues reported. A significant decrease in IL-8 levels in severe sepsis patients treated with NAC, while NAC had no effect on IL-6 levels as reported in that study [26]. Several studies evaluated the immune-modulating effects of NAC and its usage in sepsis, but the results were contradictory. Considerable amount of evidence showed that NAC administration suppressed neutrophils and macrophages activation, attenuating leukocyte–endothelial cell adhesion and inhibited TNF-α and IL-8 release [26,36-38]. Emet and co-workers reported that early NAC administration in severe sepsis did not affect cytokine levels [38,39].

In the current study, immune-modulating effect is evaluated by white blood cell count, absolute neutrophil count and serum IgM levels. In NAC group, WBC count and subsequently ANC are higher than control group which would be indicated more extensive inflammatory condition. The consistent tachycardia, elevated serum creatinine levels and higher 28 days mortality rate have observed in NAC group allegedly support these findings. IgM levels decrease from1 day through 5 in both groups. All patients are IgM deficient and NAC administration neither increase nor decrease IgM levels and it seems that no immune modulation properties are found in high doses of NAC.

Cysteine rich endogenous anti-microbial peptide HβD2 is an important component of immune system (Innate and adaptive) [20,40-43]. This study attempt to investigate whether HβD2 level can be accounted as a marker of SIRS or sepsis. In addition, the study also investigate whether NAC administration (as a cysteine source with probable immune-modulating properties) can elevate HβD2 levels (HβD2 is known as an inducible one [44,45] among other defensins). Attributed roles to HβD2 in immune system are controversial, yet it is not clear whether HβD2 is an anti-inflammatory protective mediator during sepsis or vice versa. In other study, no difference was observed between survivors and non-survivors of severe sepsis and critical illness in terms of HβD2 levels. Ex-vivo inducible expression of HβD2 by peripheral blood cells was impaired in samples from patients with severe sepsis but this was not along with low levels of HβD2, interestingly HβD2 levels were significantly higher in patients with severe sepsis compared to non-septic critically ill and healthy controls. Which according to Chen et al., NF-κB was as crucial factor for activation of HβD2 gene. In conclusion Book and coworkers noted that up-regulation of HβD2 in sepsis occurred as a result of elevated pro-inflammatory cytokines (such as IL-1 and TNF) [46,47] and was an indicator of higher activity of inflammation and the availability of sources other than peripheral blood cells for HβD2 [21].

Circulating defensins were increased in non-neutropenic children with systemic infection and sepsis. Neutrophils were the major source of defensins, they served as a specific marker for PMN activation. Defensins were not associated with organ failure or poor outcome during sepsis, defensins released into systemic circulation is an indicator of host response to sepsis. These were observations from Thomas and colleagues in a study on levels of HβD2 and lactoferrin in children with severe sepsis, the reported range by them was 450 ng/ml (median) and 194–1032 (25%-75% range) for day 1, and 300 nano-gram/ml (median) and 75–681 (25%-75% range) [41].

We hypothesized that NAC as a cysteine rich source could increase HβD2 levels and our result has confirmed this hypothesis. According to the results of our study, HβD2 levels are associated with an upward trend in NAC and a downward trend in control group. The difference between means of the two groups, however were only noteworthy on day 3. Results indicate that NAC administration with such dosage may have induced HβD2 expression. Regarding the previous discussion on immune-modulating effect of NAC, the elevated HβD2 levels in response to NAC administration appears to be the consequence of profound cysteine source. In our survey, despite higher level of HβD2 in SIRS and sepsis, no potential benefits were found concerning SOFA score and outcome (evaluated based on 28 days mortality rate and ICU length of stay). In accordance with our results supposedly considered as a marker of inflammation in patients with sepsis, however, due to limitations of our study regarding sample size, difficulties in measuring other defensin subtypes with corresponding structure and lack of data on important cytokines like IL-1 and TNF, further studies are necessary in order to clarify HβD2 and other defensin activities and characterization of potential therapeutic or diagnostic targets.

One of the possible protective effects of NAC as a preventive in contrast induced nephropathy still remains undetermined. Based on various studies and meta -analyses NAC administration decreases the risk of contrast induced nephropathy [48-52]. In addition NAC may attenuate contrast induced nephropathy, as defined by albumin excretion, and seems to be independent of any effect on creatinine (NAC dosage: 500 mg IV prior to cardiac catheterization) [53].

Marenzi et al. suggested that NAC should have a protective effect solely when oxidative stress, induced by ischemia and reperfusion, is present (NAC dosage: 600 mg intravenous bolus before primary angioplasty and 600 mg orally twice daily for the 48 hours after angioplasty) [54]. Carbonell et al. found NAC’s protect in the prevention of contrast induced nephropathy is based on reducing serum creatinine (SrCr) levels. The incidence of contrast induced nephropathy was significantly lower in NAC group but this effect was only valuable in high risk patients considering similar results that normal saline hydration produced in patients with normal renal function [55,56]. In conclusion Carbonell and coworkers recommend intravenous NAC prior to coronary angioplasty in high risk patients with SrCr ≥1.4 mg/dl, especially when hydration is impossible (NAC dosage: 600 mg every 12 hours IV) [56]. Macedo et al. in a study with focus on acute renal failure (ARF) associated with elective aortic aneurysm repair surgery, did not observe a change in ARF incidence and SrCr peak by NAC administration, in summary the study implied that potential beneficial effects of NAC in prevention of radio-contrast induced nephropathy should not be generalized to other insults applied to kidneys (NAC dosage: 1200 mg orally twice daily 24 hours before and 600 mg IV twice daily for 48 hours after) [57].

In chronic kidney disease (CKD) patients, some benefits has been observed by NAC, NAC reduces cardiovascular events in patients with stage 5 CKD and improves nitric oxide bioavailability (NAC dosage: Tepel et al., 600 mg Twice daily orally, Efrati et al. 1gr IV twice daily, 24 hours before and after angiography) [58,59]. Wittstock et al. demonstrated administration of NAC during dialysis session significantly improved arterial vascular reactivity in end stage CKD patients (NAC dosage: 5 gram IV during dialysis session) [60]. Contrary to that Renke et al. had reported, NAC had no effect on proteinuria and markers of tubular and renal fibrosis in non-diabetic CKD patients (NAC dosage: 1200 mg/day orally) [61].

In a study by Rosato and coworkers who evaluated the NAC effects on vascular system function in Systemic Sclerosis patients, authors concluded that NAC improved vascular renal function in patients with low severity of disease (NAC dosage: 15 mg/kg/hour for 5 hours) [62].

Our study was not designed to assess how NAC affects the kidneys function, not to mention the fact that the dosage of NAC administered in this study was higher than dosages administered in studies on nephro-protective properties. Considering that rising in SrCr levels is noticeable. Using paired *t*-test (before after analysis) statistical method shows a significant increase in the mean of SrCr level in NAC group while at the same time the mean level of SrCr in control group is reduced. The mean level of SrCr during NAC administration have reached 1.48 mg/dl on day 3 and interestingly SrCr levels after discontinuation of NAC (on day 3) is decreased to its baseline (as low as 0.92 mg/dl). This may partially declare that high doses NAC administration is associated with progression towards acute kidney injury.

## Conclusion

Considering adverse reactions observed in our study in terms of tachycardia, high blood pressure, leukocytosis and prolonged ventilator days and regarding the fact that no signs of improvement were observed in terms of mortality rate which was slightly higher for NAC group, high dosage of NAC is not recommended in critically ill patients with sepsis. NAC usage, even low dose, shall be administered with great caution in critically ill patients. Further studies on dosage and possible ceiling effect for NAC and re-evaluating safety of that, being conducted along with increased risk and extent of inflammatory profile. In this study no immuno-modulating effect associated with NAC was detected. According to SrCr variations during our study, we recommend to conduct further studies with adequate sample size to evaluate nephro-protective effects of NAC.

## Competing interests

The authors declare that they have no competing interests.

## Authors’ contributions

MM: study design, patient selection, sample collection and handling, statistical analysis, analysis and interpretation of data, draft the manuscript and revising. KHA: study design, patient selection, sample collection and handling, analysis and interpretation of data, draft the manuscript and revising. MA: study design, sample analyzing via commercially available enzyme-linked immunosorbent assay kit, revised the manuscript. AN: study design, patient selection, sample collection and handling, draft the manuscript and revising. MM: study design. LCK: draft the manuscript and revising. FN: interpretation of data, revising the manuscript. AA: study design, patient selection, sample collection and handling, statistical analysis, analysis and interpretation of data, draft the manuscript and revising. All authors read and approved the final manuscript.

## References

[B1] Ertel W Trentz O Polytrauma and multi-organ failure syndrome. Definition-pathophysiology-therapy Zentralbl Chir 1994 119 159 7513933

[B2] Vyrostek SB Annest JL Ryan GW Surveillance for fatal and nonfatal injuries-United States, 2001 MMWR Surveill Summ 2004 53 1 57 15343143

[B3] Bardenheuer M Obertacke U Waydhas C Nast-Kolb D Epidemiology of the severe multiple trauma-a prospective registration of preclinical and clinical supply J Orthop Trauma 2000 14 453

[B4] Esposito TJ Sanddal TL Reynolds SA Sanddal ND Effect of a voluntary trauma system on preventable death and inappropriate care in a rural state J Trauma and Acute Care Surgery 2003 54 663 670 10.1097/01.TA.0000058124.78958.6B 12707527

[B5] Brohi K Singh J Heron M Coats T Acute traumatic coagulopathy J Trauma 2003 54 1127 12813333 10.1097/01.TA.0000069184.82147.06

[B6] Homer T Peter C Frederick B Causes of death following multiple trauma Curr Orthop 2004 18 304 310

[B7] Swain SD Rohn TT Quinn MT Neutrophil priming in host defense: role of oxidants as priming agents Antioxid Redox Signal 2002 4 69 83 11970845 10.1089/152308602753625870

[B8] Martin P D’Souza D Martin J Wound healing in the PU. 1 null mouse—tissue repair is not dependent on inflammatory cells Curr Biol 2003 13 1122 1128 12842011 10.1016/s0960-9822(03)00396-8

[B9] Hietbrink F Koenderman L Rijkers G Trauma L The role of the innate immune system World J Emergency Surgery 2006 1 15 10.1186/1749-7922-1-15 PMC1481567 16759367

[B10] Power CP Wang JH Manning B Bacterial lipoprotein delays apoptosis in human neutrophils through inhibition of caspase-3 activity: regulatory roles for CD14 and TLR-2 J Immunol 2004 173 5229 5237 15470068 10.4049/jimmunol.173.8.5229

[B11] Echtenacher B Hultner L Mannel DN Cellular and molecular mechanisms of TNF protection in septic peritonitis J Inflamm 1996 47 85 89 8913934

[B12] Cobb JP O’Keefe GE Injury research in the genomic era Lancet 2004 363 2076 2083 15207961 10.1016/S0140-6736(04)16460-X

[B13] Vincent J-L Sakr Y Sprung CL Sepsis in European intensive care units: results of the SOAP study* Crit Care Med 2006 34 344 353 16424713 10.1097/01.ccm.0000194725.48928.3a

[B14] Silva E Pedro MA Sogayar A Brazilian sepsis epidemiological study (BASES study) Crit Care 2004 8 R251 R260 15312226 10.1186/cc2892 PMC522852

[B15] Martin GS Mannino DM Eaton S Moss M The epidemiology of sepsis in the United States from 1979 through 2000 N Engl J Med 2003 348 1546 1554 12700374 10.1056/NEJMoa022139

[B16] Angus DC Pereira C Silva E Epidemiology of severe sepsis around the world Endocr Metab Immune Disord Drug Targets 2006 6 207 16787296 10.2174/187153006777442332

[B17] Milner SM Bhat S Buja M Gulati S Poindexter BJ Bick RJ Expression of human β defensin 2 in thermal injury Burns 2004 30 649 654 15475136 10.1016/j.burns.2004.06.001

[B18] Burd RS Furrer JL Sullivan J Smith AL Murine [BETA]-defensin-3 is an inducible peptide with limited tissue expression and broad-spectrum antimicrobial activity Shock 2002 18 461 464 12412627 10.1097/00024382-200211000-00013

[B19] Ganz T Lehrer RI Defensins Pharmacol Ther 1995 66 191 205 7667395 10.1016/0163-7258(94)00076-f

[B20] Röhrl J Yang D Oppenheim JJ Hehlgans T Human β-defensin 2 and 3 and their mouse orthologs induce chemotaxis through interaction with CCR2 J Immunol 2010 184 6688 6694 20483750 10.4049/jimmunol.0903984 PMC6309988

[B21] Book M Chen Q Lehmann LE Inducibility of the endogenous antibiotic peptide β-defensin 2 is impaired in patients with severe sepsis Crit Care 2007 11 R19 17302973 10.1186/cc5694 PMC2151902

[B22] Millea PJ N-acetylcysteine: multiple clinical applications Am Fam Physician 2009 80 265 269 19621836

[B23] Kelly GS Clinical applications of N-acetylcysteine. Alternative medicine review J Clinical Therapeutic 1998 3 114 9577247

[B24] Atkuri KR Mantovani JJ Herzenberg LA N-Acetylcysteine-a safe antidote for cysteine/glutathione deficiency Curr Opin Pharmacol 2007 7 355 359 17602868 10.1016/j.coph.2007.04.005 PMC4540061

[B25] Santiago F Bueno P Olmedo C Effect of n-Acetylcysteine administration on intraoperative plasma levels of interleukin-4 and interleukin-10 in liver transplant recipients Transplant Proc 2008 40 2978 2980 19010165 10.1016/j.transproceed.2008.08.103

[B26] Paterson RL Galley HF Webster NR The effect of N-acetylcysteine on nuclear factor-[kappa] B activation, interleukin-6, interleukin-8, and intercellular adhesion molecule-1 expression in patients with sepsis* Crit Care Med 2003 31 2574 2578 14605526 10.1097/01.CCM.0000089945.69588.18

[B27] Heller AR Groth G Heller SC N-acetylcysteine reduces respiratory burst but augments neutrophil phagocytosis in intensive care unit patients Crit Care Med 2001 29 272 276 11246305 10.1097/00003246-200102000-00009

[B28] Lee TF Jantzie LL Todd KG Cheung PY Postresuscitation N-acetylcysteine treatment reduces cerebral hydrogen peroxide in the hypoxic piglet brain Intensive Care Med 2008 34 190 197 17938888 10.1007/s00134-007-0880-z

[B29] Molnar Z MacKinnon K Shearer E Lowe D Watson I The effect of N-acetylcysteine on total serum anti-oxidant potential and urinary albumin excretion in critically ill patients Intensive Care Med 1998 24 230 235 9565804 10.1007/s001340050555

[B30] Molnar Z Shearer E Lowe D N-Acetylcysteine treatment to prevent the progression of multisystem organ failure: A prospective, randomized, placebo-controlled study Crit Care Med 1999 27 1100 1104 10397212 10.1097/00003246-199906000-00028

[B31] Spies CD Reinhart K Witt I Influence of N-acetlcycteine on direct indicators of tissue oxygenation in septicshock patients: Results from a prospective, randomized, double-blind study Crit Care Med 1994 22 1738 1746 7956276

[B32] Ortolani O Conti A De Gaudio AR Protective effects of N-acetlcycteine and rutin on the lipid peroxidation of the lung epithelium during the adult respiratory syndrome Shock 2000 13 14 18 10638663 10.1097/00024382-200013010-00003

[B33] Szakmany T Marton S Molnar Z Lack of effect of prophylactic N-acetlcycteine on postopertavie organdysfunction following major abdominal tumour surgery Anaesth Intensive Care 2003 31 267 271 12879670 10.1177/0310057X0303100304

[B34] Domenighetti G Suter PM Schaller MD Ritz R Perret C Treatment with N -acetylcysteine during acute respiratory distress syndrome: a randomized, double-blind, placebo-controlled clinical study J Crit Care 1997 12 177 182 9459113 10.1016/s0883-9441(97)90029-0

[B35] Bernard GR Wheeler AP Arons MM A trial of antioxidants N-acetylcysteine and procysteine in ARDS Chest 1997 112 164 172 9228372 10.1378/chest.112.1.164

[B36] Spapen H N-acetylcysteine in clinical sepsis: a difficult marriage Crit Care 2004 8 229 230 15312203 10.1186/cc2887 PMC522849

[B37] Schmidt H Schmidt W Muller T Bohrer H Gebhard MM Martin E N-acetylcysteine attenuates endotoxin-induced leukocyte-endothelial cell adhesion and macromolecular leakage in vivo Crit Care Med 1997 25 858 863 9187607 10.1097/00003246-199705000-00023

[B38] Kharazmi A Nielsen H Schiøtz P N-acetylcysteine inhibits human neutrophil and monocyte chemotaxis and oxidative metabolism Int J Immuno Pharmacology 1988 10 39 46 10.1016/0192-0561(88)90148-8 3366508

[B39] Emet S Memis D Pamukçu Z The influence of N-acetyl-L-cystein infusion on cytokine levels and gastric intramucosal pH during severe sepsis Crit Care 2004 8 229 230 15312215 10.1186/cc2866 PMC522835

[B40] Yamaguchi Y Ouchi Y Antimicrobial peptide defensin: Identification of novel isoforms and the characterization of their physiological roles and their significance in the pathogenesis of diseases Proc Jpn Acad Ser B Phys Biol Sci 2012 88 152 10.2183/pjab.88.152 PMC3406309 22498979

[B41] Thomas NJ Carcillo JA Doughty LA Sasser H Heine RP Plasma concentrations of defensins and lactoferrin in children with severe sepsis Pediatr Infect Dis J 2002 21 34 38 11791096 10.1097/00006454-200201000-00008

[B42] Shi Z Zhao Z Shu Q Protective effect of recombinant beta-defensin-2 on acute lung injury induced by sepsis in rats Zhejiang da xue xue bao Yi xue ban J Zhejiang University Medical sciences 2006 35 605 10.3785/j.issn.1008-9292.2006.06.006 17177331

[B43] Krisanaprakornkit S Kimball JR Weinberg A Darveau RP Bainbridge BW Dale BA Inducible expression of human β-defensin 2 byFusobacterium nucleatum in oral epithelial cells: multiple signaling pathways and role of commensal bacteria in innate immunity and the epithelial barrier Infect Immun 2000 68 2907 2915 10768988 10.1128/iai.68.5.2907-2915.2000 PMC97503

[B44] Harder J Bartels J Christophers E Schroder J A peptide antibiotic from human skin Nature 1997 387 861 61 9202117 10.1038/43088

[B45] Liu K-X Chen S-Q Zhang H Guo J-y Li Y-S Huang W-Q Intestinal ischaemia/reperfusion upregulates β-defensin-2 expression and causes acute lung injury in the rat Injury 2009 40 950 955 19486970 10.1016/j.injury.2009.01.103

[B46] Tsutsumi-Ishii Y Nagaoka I Modulation of human β-defensin-2 transcription in pulmonary epithelial cells by lipopolysaccharide-stimulated mononuclear phagocytes via proinflammatory cytokine production J Immunol 2003 170 4226 4236 12682256 10.4049/jimmunol.170.8.4226

[B47] Liu L Roberts AA Ganz T By IL-1 signaling, monocyte-derived cells dramatically enhance the epidermal antimicrobial response to lipopolysaccharide J Immunol 2003 170 575 580 12496445 10.4049/jimmunol.170.1.575

[B48] Tepel M Van Der Giet M Schwarzfeld C Laufer U Liermann D Zidek W Prevention of radiographic-contrast-agent–induced reductions in renal function by acetylcysteine N Engl J Med 2000 343 180 184 10900277 10.1056/NEJM200007203430304

[B49] Kay J Chow WH Chan TM Acetylcysteine for prevention of acute deterioration of renal function following elective coronary angiography and intervention JAMA 2003 289 553 558 12578487 10.1001/jama.289.5.553

[B50] Durham JD Caputo C Dokko J A randomized controlled trial of N-acetylcysteine to prevent contrast nephropathy in cardiac angiography Kidney Int 2002 62 2202 2207 12427146 10.1046/j.1523-1755.2002.00673.x

[B51] Duong MH MacKenzie TA Malenka D N‒acetylcysteine prophylaxis significantly reduces the risk of radiocontrast‒induced nephropathy: comprehensive meta‒analysis Catheter Cardiovasc Interv 2005 64 471 479 15789388 10.1002/ccd.20342

[B52] Birck R Krzossok S Markowetz F Schnülle P Van Der Woude FJ Braun C Acetylcysteine for prevention of contrast nephropathy: meta-analysis Lancet 2003 362 598 603 12944058 10.1016/S0140-6736(03)14189-X

[B53] Levin A Pate GE Shalansky S N-acetylcysteine reduces urinary albumin excretion following contrast administration: evidence of biological effect Nephrol Dial Transplant 2007 22 2520 2524 17557777 10.1093/ndt/gfl707

[B54] Marenzi G Assanelli E Marana I N-acetylcysteine and contrast-induced nephropathy in primary angioplasty N Engl J Med 2006 354 2773 2782 16807414 10.1056/NEJMoa054209

[B55] Miner SE Dzavik V Nguyen-Ho P N-acetylcysteine reduces contrast-associated nephropathy but not clinical events during long-term follow-up Am Heart J 2004 148 690 695 15459602 10.1016/j.ahj.2004.05.015

[B56] Carbonell N Sanjuán R Blasco M Jordá Á Miguel A N-acetylcysteine: short-term clinical benefits after coronary angiography in high-risk renal patients Rev Esp Cardiol 2010 63 12 19 20089221 10.1016/s1885-5857(10)70004-9

[B57] Macedo E Abdulkader R Castro I Sobrinho AC Yu L Vieira JM Lack of protection of N-acetylcysteine (NAC) in acute renal failure related to elective aortic aneurysm repair-a randomized controlled trial Nephrol Dial Transplant 2006 21 1863 1869 16522657 10.1093/ndt/gfl079

[B58] Tepel M van der Giet M Statz M Jankowski J Zidek W The antioxidant acetylcysteine reduces cardiovascular events in patients with End-stage renal failure a randomized, controlled trial Circulation 2003 107 992 995 12600912 10.1161/01.cir.0000050628.11305.30

[B59] Efrati S Dishy V Averbukh M The effect of N-acetylcysteine on renal function, nitric oxide, and oxidative stress after angiography Kidney Int 2003 64 2182 2187 14633141 10.1046/j.1523-1755.2003.00322.x

[B60] Wittstock A Burkert M Zidek W Tepel M Scholze A N-acetylcysteine improves arterial vascular reactivity in patients with chronic kidney disease Nephron Clin Pract 2009 112 c184 c189 19439989 10.1159/000218107

[B61] Renke M Tylicki L Rutkowski P The effect of N-acetylcysteine on proteinuria and markers of tubular injury in Non-diabetic patients with chronic kidney disease Kidney Blood Press Res 2008 31 404 410 19092257 10.1159/000185828

[B62] Rosato E Cianci R Barbano B N-acetylcysteine infusion reduces the resistance index of renal artery in the early stage of systemic sclerosis Acta Pharmacol Sin 2009 30 1283 1288 19730428 10.1038/aps.2009.128 PMC4007187

